# Gliomatosis Cerebri: A Unique Presentation with Accompanying Clinical Nuance

**DOI:** 10.7759/cureus.5149

**Published:** 2019-07-16

**Authors:** Christ Ordookhanian, Ryan F Amidon, Paul E Kaloostian

**Affiliations:** 1 Miscellaneous, School of Medicine, University of California, Riverside, USA; 2 Neuroscience, University of California, Riverside, USA; 3 Neurological Surgery, Paul Kaloostian, M.D., Inc., Riverside, USA

**Keywords:** glioma, gliomatosis cerebri, cancer, histology, astrocyte

## Abstract

Gliomatosis cerebri (GC) has classically been considered a rare malignancy with a poor prognosis and is developmentally unique from solid tumors. More recently, GC has become better understood as a phenotype along the spectrum of gliomas and, most importantly, not mutually exclusive from the more common presentation of a tumor mass. The following case report illustrates not only the implications of the ontogeny of gliomas in clinical practice but also the successes that can accompany the early recognition of such a disease. Here, we report the presentation of a solid temporal lobe glioma, which, on presentation, was disseminating along well-defined mesolimbic white matter tracts. Once properly diagnosed and managed, the patient remarkably proceeded to achieve an impressive outcome given the extent of her pathology.

## Introduction

Gliomatosis cerebri (GC) is a rare and rapidly infiltrating brain tumor of the glial family of brain tumors for which prognosis is rather dismal. Typically, within one year of the symptomatic onset of GC, patient survivability ranges between 25% and 50% [1]. Since the inception of the categorical ranking of GC within the glioma cancer family, pathophysiology and epidemiology have been rather scarce in data, resulting in a World Health Organization (WHO) classification of GC as a pattern of neoplastic cell distribution upon histopathological sampling [2]. A 2017 manuscript from the National Cancer Institute by Ranjan et al. described that the pathology of GC as a “grand manifestation of diffuse glioma,” further highlighting the degree of uncertainty associated with the GC pathology as well as its classification outside of histological studies, which may be challenging clinically [3]. Currently, GC has been identified to satisfy two categories, primary GC, which is de novo with no obvious mass present, and secondary GC, where a diffuse infiltrative pattern is present and associated with a neoplastic tumor mass [4]. Per the work of Chen et al. in a 2013 study, it was identified that the GC pathology presenting in patients over the age of 46, as well as in patients with a higher histopathological grading upon a pathology review, showed decreased overall survival. Therapeutically, the literature indicates that the predominant first-line attack against this aggressive, vastly proliferative, glioma is radiation therapy, which was associated with positive prognosis and increased survivability while chemotherapy, per study results, is contraindicated and associated with poor prognosis [5]. Upon determination of surgical candidacy, patients may also be required to undergo partial resection of additional cephalic regions that appear abnormal on magnetic resonance imaging (MRI) with minimization of iatrogenic outcomes [3].

## Case presentation

**Figure 1:**
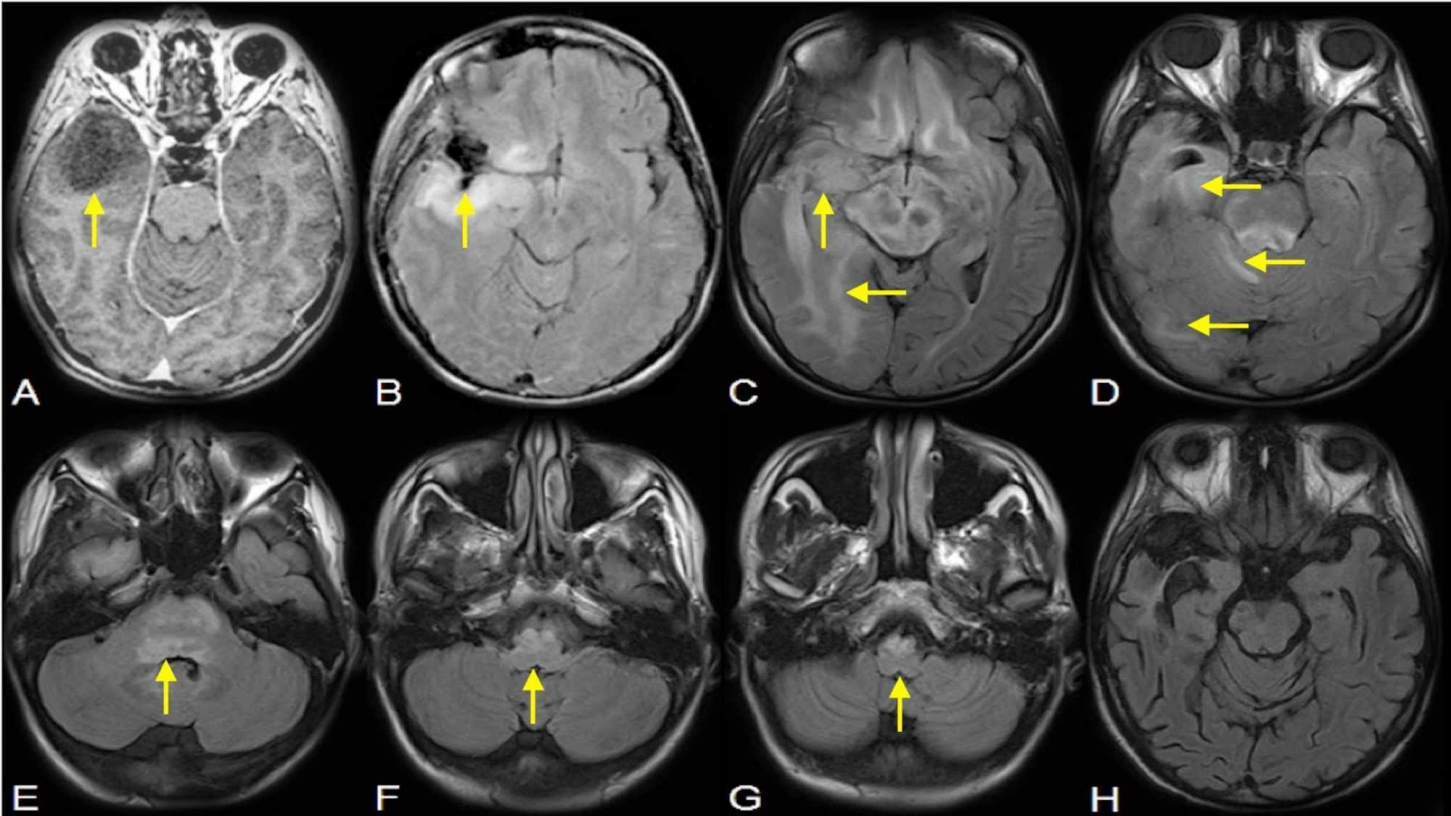
Gliomatosis Cerebri Radiographic Representation (A) Preoperative T1 postgadolinium magnetic resonance (MR) image showing right anterior temporal lobe mass. (B) Post-operative fluid-attenuated inversion recovery (FLAIR) image showing a residual tumor, in retrospect. (C-G) At 45 months follow-up, the lesion is now an obvious case of gliomatosis cerebri with tumor spread following multi-lobar mesolimbic system functional connections as well as both bilateral supratentorial and brain stem involvement. (H) Significant post-treatment diminution of multicompartmental mesolimbic gliomatosis with near-complete resolution of FLAIR hyperintensity in deeper brainstem structures thought to be compromised in central neurogenic hyperventilation (CNH).

We report a 24-year-old, right-handed woman presenting upon referral from emergency medicine (EM) for the evaluation of a possible recurrence of temporal lobe astrocytoma (TLA). The patient initially presented at the age of 21 with an eight-month history of partial-complex seizures. MRI revealed a non-enhancing fluid attenuation inversion recovery imaging (FLAIR) predominant lesion in the right temporal lobe subtly extending into the right orbital frontal cortex and insula (Figure 1A). Anterior temporal lobectomy was performed revealing a World Health Organization (WHO) Grade II astrocytoma. Unfortunately, the seizures returned after 10 months. Post-operative serial MRI studies showed continuing FLAIR/T2 hyperintensity in the right frontal cortex and insular areas expanding along well-known mesolimbic pathways (Figure 1B). These findings were unfortunately labeled as post-surgical changes until the patient moved to California and was referred to our multidisciplinary brain tumor program for re-evaluation.

The patient delayed planned involved-field radiotherapy and did not follow up with us for an additional four months, at which time she was admitted for dehydration and found to have severe metabolic alkalosis with an arterial blood gas pH of 7.65, arterial partial pressure of carbon dioxide (P_a_CO_2_) of 12.7 mmHg, arterial partial pressure of oxygen (P_a_O_2_) of 138.9 mm Hg, concentration of bicarbonate (HCO_3_) of 13.8 mEq/L, a base deficit of -3 mEq/L, and hemoglobin oxygen saturation (S_a_O_2_) of 99.7%.

MRI showed a massive progression of her disease, with Gliomatosis cerebri (GC) involving the medial basal structures of both frontal lobes, right insula, midbrain, pons, right middle cerebellar peduncle, and medulla (Figures 1C-1G). The patient was admitted to the intensive care unit (ICU) for the management of central neurogenic hyperventilation (CNH). Her course was complicated by hospital-acquired pneumonia, thrombocytopenia, transaminitis, hypothyroidism, persistent hypotension, pseudomonas bacteremia, and a vancomycin-resistant enterococcus urinary tract infection. She was treated aggressively in accordance with family wishes and kept intubated through six weeks of radio-chemotherapy in addition to the treatment of her medical conditions.

She made a remarkable recovery after the conclusion of radiotherapy and continued her treatment with temozolomide (Figure 1H). She was eventually discharged to her parents’ home, with her Karnofsky performance status (KPS), a standardized measuring tool of a patients' ability to perform ordinary tasks, having increased from 20 to 70 but with an eventual decrease only 12 months later due to a new episode of tumor progression.

## Discussion

Gliomatosis cerebri is a rare neoplasm characterized by diffuse invasion along white matter tracts and blood vessels. Median survival is approximately 12-14 months. Classically, GC has been defined as involving at least two lobes of the brain and has been thought to be distinct from focal, solid glial tumors [6]. More recently, GC has become better understood as an indicative individual cell invasive or migratory phenotype over proliferative, angiogenic, solid tumor-mass phenotype. While the former is becoming better understood as a major factor negatively influencing prognosis and survival, the latter is a major factor in patient symptomatology [7-8]. Unfortunately, the molecular mechanisms underlying glioma invasion and solid tumor growth have not yet been definitively elucidated, and many continue to view gliomas based purely on the classic description of solid tumor, light microscope-based classification.

In retrospect, initial imaging in our patient fit criteria for both a solid tumor and gliomatosis cerebri. Her imaging studies clearly showed a solid temporal lobe tumor in addition to an invasive component extending not only along the insula but also into the right frontal lobe via the diagonal band of Broca. While it could be argued at the time of surgery that this represented peri-tumoral edema, the continued progression of this lesion along defined mesolimbic pathways should have signaled the presence of GC and led to aggressive treatment. Instead, the patient went without further treatment for roughly 45 months. At the time of admission, she had diffuse bilateral involvement, including the brainstem, leading to interference with well-described respiratory brainstem centers manifesting as CNH. Her CNH did not resolve until after the completion of radiotherapy and the radiographic improvement of her brainstem disease [9-10]. Aggressive treatment resulted in significant functional recovery and increased the patient’s survival by an additional year. With advances in a wide array of disciplines, including genomics, proteomics, chemotherapy, and neuroimaging, a comprehensive picture of tumor biological expression profiles is slowly emerging [11-12]. While often challenging established concepts of tumor characterization and classification, these advances hold the exciting potential of leading to improved treatments.

## Conclusions

This case, in particular, illustrates the importance of appreciating the nuances of solid and non-solid appearing invasive tumors with respect to the earliest diagnosis of GC, the potential for GC to present with CNH, and the ability of aggressive multidisciplinary critical care and oncological therapy to successfully treat this condition. This case is extremely rare due to the age presentation and the aggressive proliferation of an ultimately lethal pathology that took on many proliferative avenues into vital brain centers for which radiation therapy and chemotherapy did not vastly change prognostic outcomes. In addition, this case highlights the crucial component of time in the proper management of all aggressive neoplasms, including GC.
